# Supplementary material for: Construction of Metastasis Prediction Models and Screening of Anti-Metastatic Drugs Based on Pan-Cancer Single-Cell EMT Features
Source: Int J Mol Sci. 2025 Nov 29;26(23):11582. doi: 10.3390/ijms262311582 (PMC12692492; doi:10.3390/ijms262311582)
Supplement: Supplementary file 1 [file ijms-26-11582-s001.zip › Additional file S1.pdf]

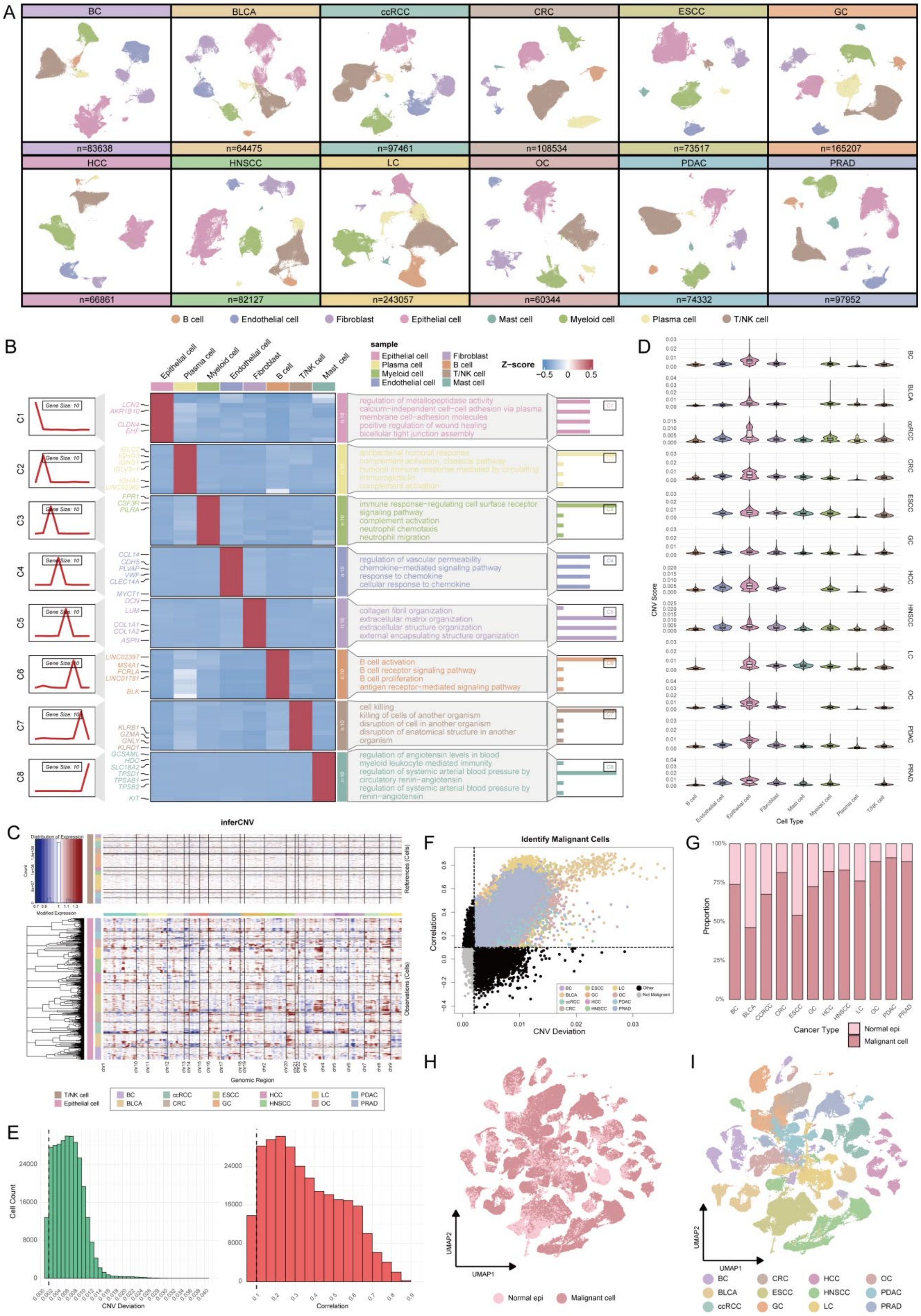
A
BC
BLCA
ccRCC
CRC
ESCC
GC
n=83638
n=64475
n=97461
n=108534
n=73517
n=165207
HCC
HNSCC
LC
OC
PDAC
PRAD
n=66861
n=82127
n=243057
n=60344
n=74332
n=97952
B cell
Endothelial cell
Fibroblast
Epithelial cell
Mast cell
Myeloid cell
Plasma cell
T/NK cell
B
sample
Z-score
C1
C2
C3
C4
C5
C6
C7
C8
Gene Size: 10
LCN2
AKR1B10
CLDN4
EHF
FPR1
CSF3R
PILRA
CCL14
CDH5
PLVAP
VWF
CLEC14A
MYCT1
DCN
LUM
COL1A1
COL1A2
ASPN
LINC02397
MS4A1
FCRLA
LINC01781
BLK
KLRB1
GZMA
GNLY
KLRD1
GCSAML
HDC
SLC18A2
TPSD1
TPSAB1
TPSB2
KIT
regulation of metallopeptidase activity
calcium−independent cell−cell adhesion via plasma
membrane cell−adhesion molecules
positive regulation of wound healing
bicellular tight junction assembly
antibacterial humoral response
complement activation, classical pathway
humoral immune response mediated by circulating
immunoglobulin
complement activation
immune response−regulating cell surface receptor
signaling pathway
complement activation
neutrophil chemotaxis
neutrophil migration
regulation of vascular permeability
chemokine−mediated signaling pathway
response to chemokine
cellular response to chemokine
collagen fibril organization
extracellular matrix organization
extracellular structure organization
external encapsulating structure organization
B cell activation
B cell receptor signaling pathway
B cell proliferation
antigen receptor−mediated signaling pathway
cell killing
killing of cells of another organism
disruption of cell in another organism
disruption of anatomical structure in another
organism
regulation of angiotensin levels in blood
myeloid leukocyte mediated immunity
regulation of systemic arterial blood pressure by
circulatory renin−angiotensin
regulation of systemic arterial blood pressure by
renin−angiotensin
D
CNV Score
Cell Type
C
inferCNV
Distribution of Expression
Modified Expression
References (Cells)
Observations (Cells)
Genomic Region
E
Cell Count
CNV Deviation
Correlation
F
Identify Malignant Cells
Correlation
CNV Deviation
Other
Not Malignant
G
Proportion
Cancer Type
Normal epi
Malignant cell
H
UMAP1
UMAP2
Normal epi
Malignant cell
I
UMAP1
UMAP2

Figure S1. Single-cell annotation across multiple cancer types and identification of malignant epithelial cells. (A) UMAP visualization of cell type classification across 12 cancer types. (B) Integrative visualization of cell type-specific features. The line plot (left) shows the expression trends of canonical marker genes across cell populations; the heatmap (middle) displays the average expression levels of the top 10 differentially expressed genes in each cell type; the bar plot (right) presents enriched GO biological processes (BP), with colors indicating distinct cell types and bar length representing enrichment significance. (C) Single-cell CNV heatmap illustrating copy number variation patterns in epithelial cells versus T/NK cells (red indicates amplification, blue indicates deletion). (D) Boxplots and violin plots showing CNV Deviation across cell types in different cancers. (E) Histogram of epithelial cells showing CNV Deviation (left, threshold = 0.002) and correlation values (right, threshold = 0.1). (F) Scatter plot for malignant epithelial cell identification, where the x-axis represents CNV Deviation and the y-axis represents correlation coefficients; red dots denote malignant cells, gray dots denote non-malignant cells, and dashed lines indicate classification thresholds. (G) Stacked bar plot showing the proportions of normal and malignant epithelial cells across cancer types. (H) UMAP visualization of the distribution of malignant versus non-malignant epithelial cells. (I) UMAP visualization showing the distribution of epithelial cells across different cancer types.

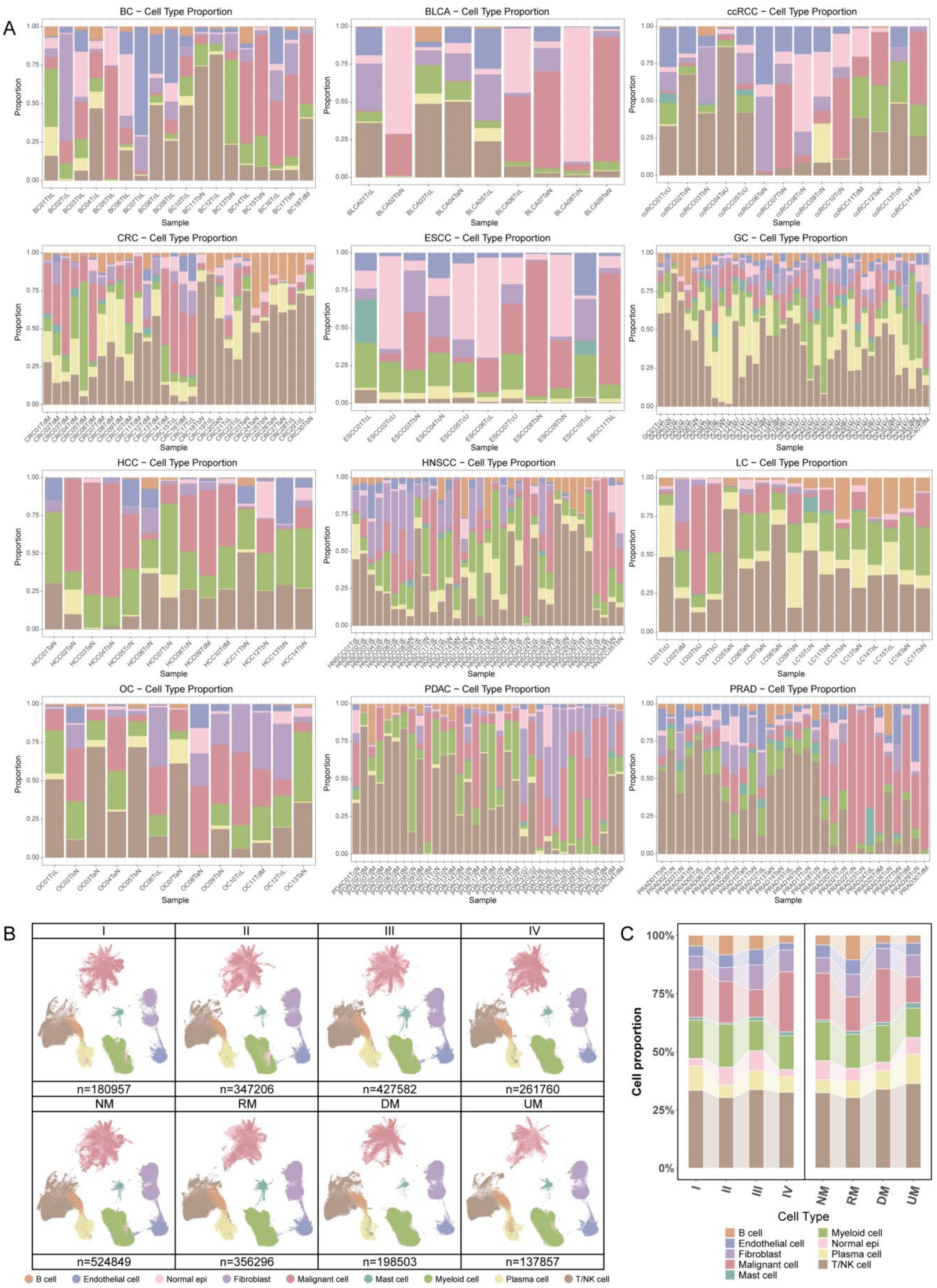
A
BC - Cell Type Proportion
BLCA - Cell Type Proportion
ccRCC - Cell Type Proportion
CRC - Cell Type Proportion
ESCC - Cell Type Proportion
GC - Cell Type Proportion
HCC - Cell Type Proportion
HNSCC - Cell Type Proportion
LC - Cell Type Proportion
OC - Cell Type Proportion
PDAC - Cell Type Proportion
PRAD - Cell Type Proportion
Proportion
Sample
B
I
II
III
IV
n=180957
n=347206
n=427582
n=261760
NM
RM
DM
UM
n=524849
n=356296
n=198503
n=137857
B cell
Endothelial cell
Normal epi
Fibroblast
Malignant cell
Mast cell
Myeloid cell
Plasma cell
T/NK cell
C
Cell proportion
100%
75%
50%
25%
0%
I
II
III
IV
NM
RM
DM
UM
Cell Type
B cell
Endothelial cell
Fibroblast
Malignant cell
Mast cell
Myeloid cell
Normal epi
Plasma cell
T/NK cell

Figure S2. Distribution characteristics of distinct cell populations in the tumor microenvironment. (A) Stacked bar plots showing the distribution of cell types across individual samples in different cancer types. (B) UMAP visualization showing cell type identification across different tumor stages and metastatic states. (C) Stacked bar plots displaying the proportions of cell types under varying tumor stages and metastatic conditions.

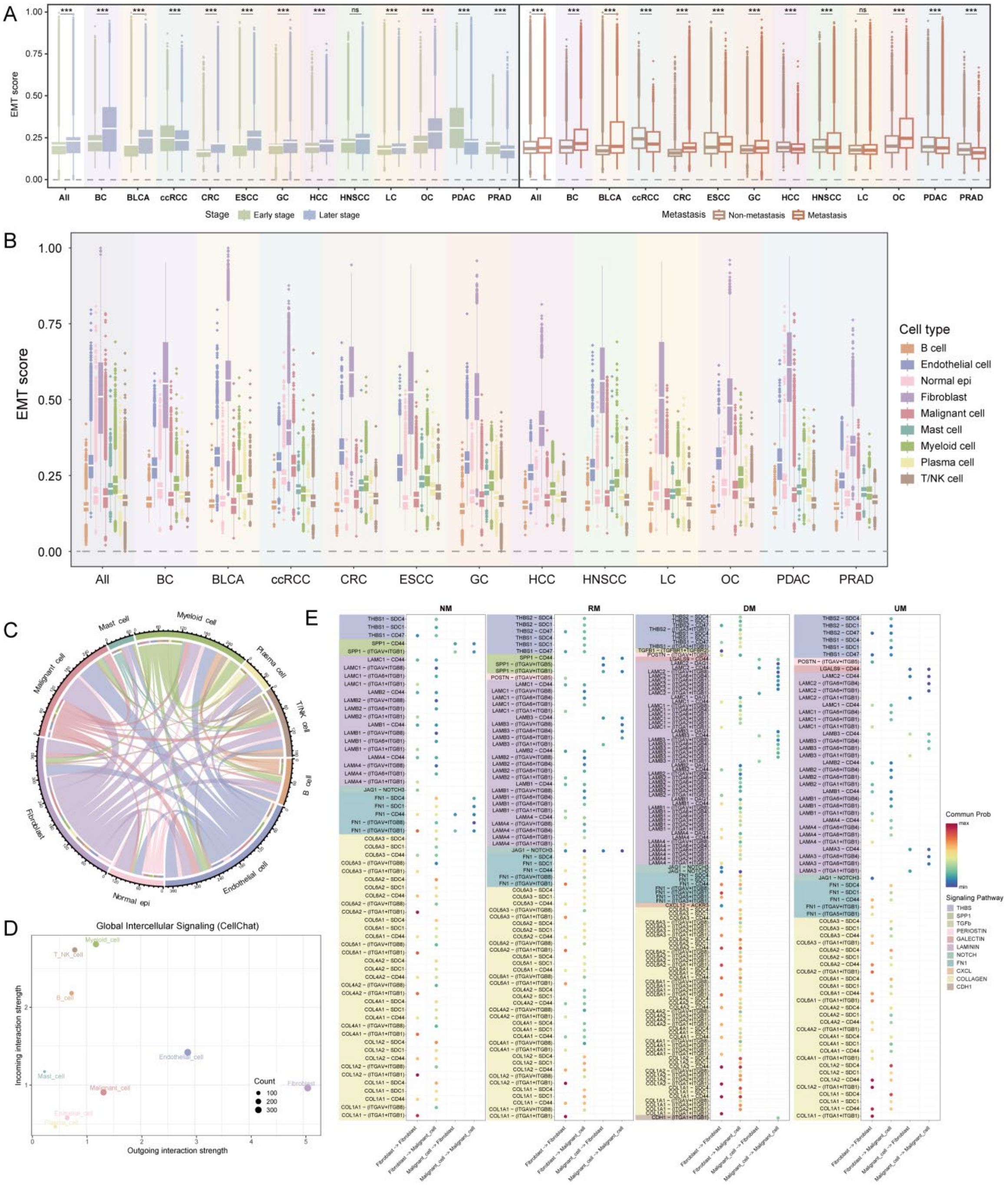

Figure S3. EMT and interaction characteristics of distinct cell populations in the tumor microenvironment. (A) Boxplots showing EMT signature scores across cancer stages and metastatic states in all cells. (B) Grouped boxplots illustrating EMT scores of different cell types across cancer types. (C) Chord diagram depicting intercellular interactions, with chord thickness representing the number of interacting pairs. (D) Scatter plot showing the roles of cell types in signal sending and receiving; node color denotes cell type and node size reflects network centrality. (E) Grouped dot plot illustrating the interaction strength between malignant epithelial cells and

fibroblasts under different metastatic states, with the blue-to-red gradient representing increasing interaction intensity.

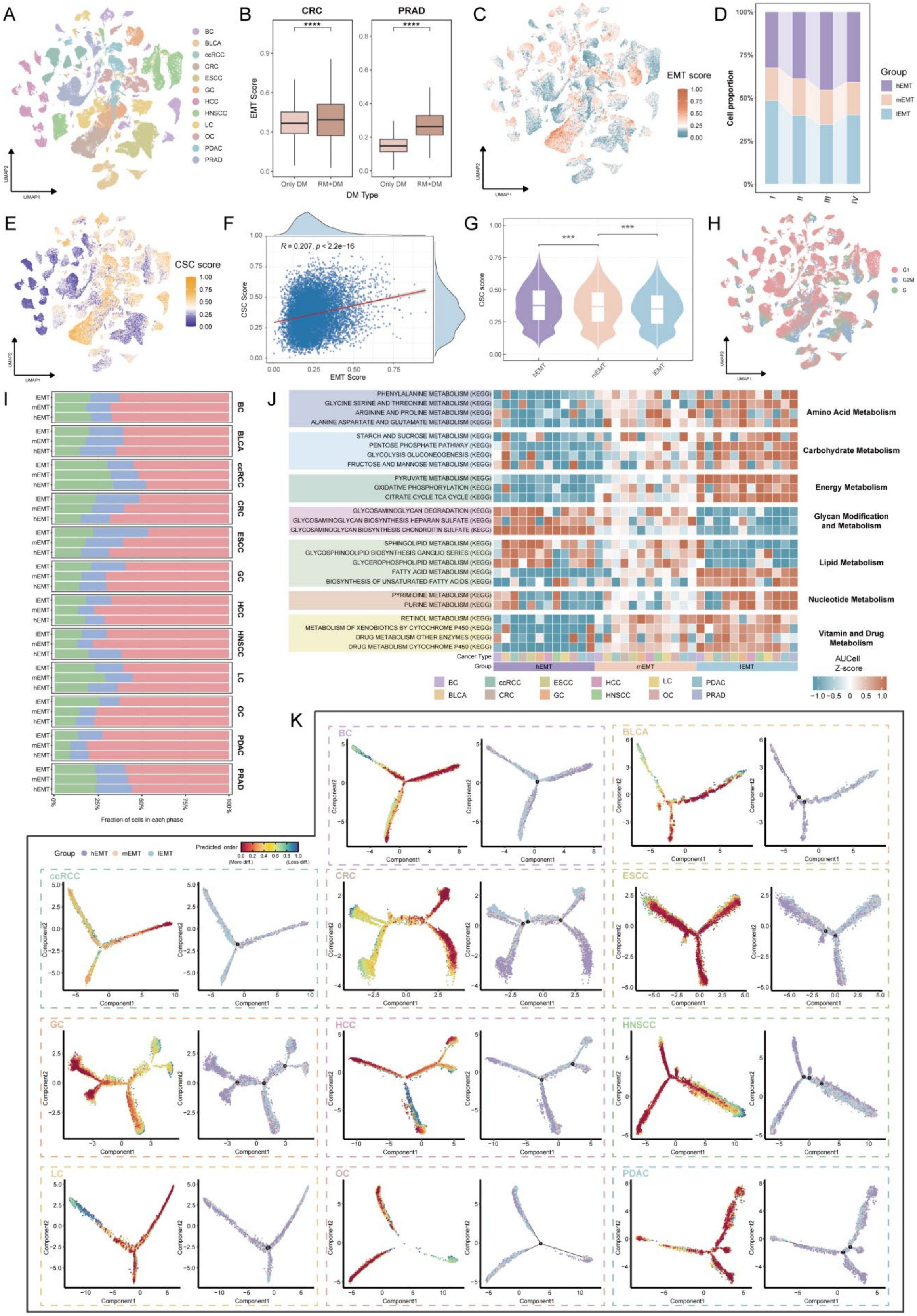
A
BC
BLCA
ccRCC
CRC
ESCC
GC
HCC
HNSCC
LC
OC
PDAC
PRAD
UMAP1
UMAP2
B
CRC
PRAD
EMT Score
Only DM
RM+DM
DM Type
C
EMT score
D
Cell proportion
Group
hEMT
mEMT
lEMT
I
II
III
IV
E
CSC score
F
R = 0.207, p < 2.2e−16
CSC Score
EMT Score
G
CSC score
H
G1
G2M
S
I
Fraction of cells in each phase
J
PHENYLALANINE METABOLISM (KEGG)
GLYCINE SERINE AND THREONINE METABOLISM (KEGG)
ARGININE AND PROLINE METABOLISM (KEGG)
ALANINE ASPARTATE AND GLUTAMATE METABOLISM (KEGG)
STARCH AND SUCROSE METABOLISM (KEGG)
PENTOSE PHOSPHATE PATHWAY (KEGG)
GLYCOLYSIS GLUCONEOGENESIS (KEGG)
FRUCTOSE AND MANNOSE METABOLISM (KEGG)
PYRUVATE METABOLISM (KEGG)
OXIDATIVE PHOSPHORYLATION (KEGG)
CITRATE CYCLE TCA CYCLE (KEGG)
GLYCOSAMINOGLYCAN DEGRADATION (KEGG)
GLYCOSAMINOGLYCAN BIOSYNTHESIS HEPARAN SULFATE (KEGG)
GLYCOSAMINOGLYCAN BIOSYNTHESIS CHONDROITIN SULFATE (KEGG)
SPHINGOLIPID METABOLISM (KEGG)
GLYCOSPHINGOLIPID BIOSYNTHESIS GANGLIO SERIES (KEGG)
GLYCEROPHOSPHOLIPID METABOLISM (KEGG)
FATTY ACID METABOLISM (KEGG)
BIOSYNTHESIS OF UNSATURATED FATTY ACIDS (KEGG)
PYRIMIDINE METABOLISM (KEGG)
PURINE METABOLISM (KEGG)
RETINOL METABOLISM (KEGG)
METABOLISM OF XENOBIOTICS BY CYTOCHROME P450 (KEGG)
DRUG METABOLISM OTHER ENZYMES (KEGG)
DRUG METABOLISM CYTOCHROME P450 (KEGG)
Cancer Type
Group
Amino Acid Metabolism
Carbohydrate Metabolism
Energy Metabolism
Glycan Modification and Metabolism
Lipid Metabolism
Nucleotide Metabolism
Vitamin and Drug Metabolism
AUCell Z-score
K
Group
Predicted order
(More diff.)
(Less diff.)
Component1
Component2

Figure S4. Group-specific characteristics and developmental trajectories of malignant epithelial cells. (A) UMAP visualization showing the distribution of malignant epithelial cells across cancer types. (B) The boxplots depict the distribution of EMT scores in two types of distant metastasis samples in CRC and PRAD. One group comprises only direct distant metastasis samples (Only DM), while the other comprises sequential lymph node and distant metastasis samples (RM+DM).(C) UMAP projection depicting the distribution of EMT signature scores derived from 200 genes within malignant epithelial cells. (D) Stacked bar plot displaying the proportions of cell types across cancer stages. (E) UMAP visualization showing the distribution of CSC signature scores derived from 8 genes in malignant epithelial cells. (F) Scatter plot showing the relationship between EMT and CSC scores after partial removal of extreme cells, with a linear regression line (red) and Pearson correlation indicated. Marginal density plots display the distributions of EMT and CSC scores. (G) Boxplots and violin plots comparing CSC signature scores across different subgroups. (H) Stacked bar plot showing the proportions of cell-cycle phases across subgroups in different cancers. (I) UMAP projection illustrating the distribution of malignant epithelial cells across distinct cell-cycle phases (G1, G2M, and S). (J) Heatmap illustrating AUCell Z-score activities of functional pathways across subgroups, with pathways hierarchically clustered by metabolic functions. (K) Developmental trajectories of malignant epithelial cells in 11 cancer types (excluding PRAD). For each cancer type, CytoTRACE plots (left) indicate predicted differentiation potential (red = low, blue = high), and Monocle trajectories (right) display lineage progression, with numbers denoting key branch points.

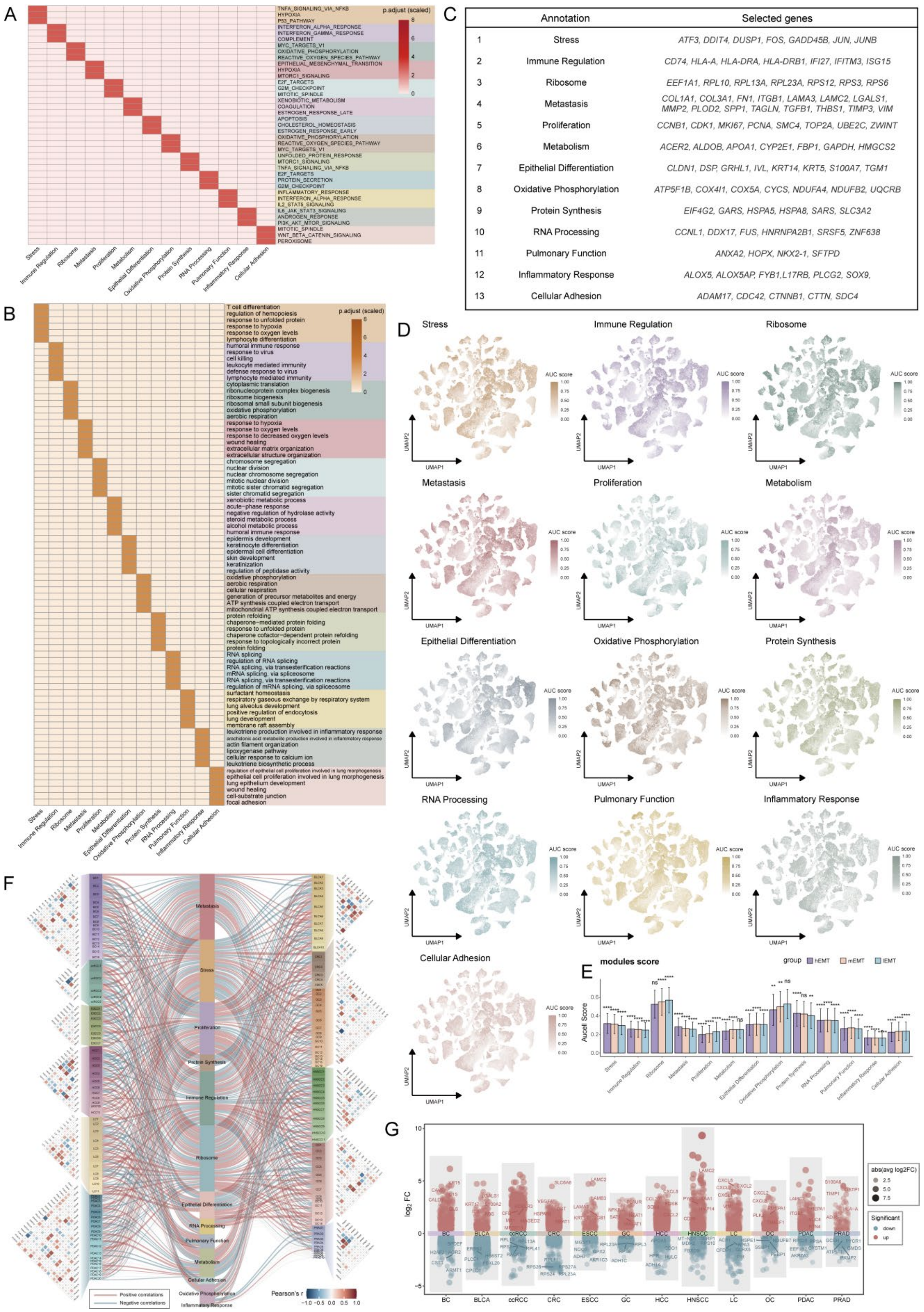
A
B
C
Annotation | Selected genes
1 Stress | ATF3, DDIT4, DUSP1, FOS, GADD45B, JUN, JUNB
2 Immune Regulation | CD74, HLA-A, HLA-DRA, HLA-DRB1, IFI27, IFITM3, ISG15
3 Ribosome | EEF1A1, RPL10, RPL13A, RPL23A, RPS12, RPS3, RPS6
4 Metastasis | COL1A1, COL3A1, FN1, ITGB1, LAMA3, LAMC2, LGALS1, MMP2, PLOD2, SPP1, TAGLN, TGFB1, THBS1, TIMP3, VIM
5 Proliferation | CCNB1, CDK1, MKI67, PCNA, SMC4, TOP2A, UBE2C, ZWINT
6 Metabolism | ACER2, ALDOB, APOA1, CYP2E1, FBP1, GAPDH, HMGCS2
7 Epithelial Differentiation | CLDN1, DSP, GRHL1, IVL, KRT14, KRT5, S100A7, TGM1
8 Oxidative Phosphorylation | ATP5F1B, COX4I1, COX5A, CYCS, NDUFA4, NDUFB2, UQCRB
9 Protein Synthesis | EIF4G2, GARS, HSPA5, HSPA8, SARS, SLC3A2
10 RNA Processing | CCNL1, DDX17, FUS, HNRNPA2B1, SRSF5, ZNF638
11 Pulmonary Function | ANXA2, HOPX, NKX2-1, SFTPD
12 Inflammatory Response | ALOX5, ALOX5AP, FYB1,L17RB, PLCG2, SOX9,
13 Cellular Adhesion | ADAM17, CDC42, CTNNB1, CTTN, SDC4
D
Stress
Immune Regulation
Ribosome
Metastasis
Proliferation
Metabolism
Epithelial Differentiation
Oxidative Phosphorylation
Protein Synthesis
RNA Processing
Pulmonary Function
Inflammatory Response
Cellular Adhesion
AUC score
UMAP1
UMAP2
E
modules score
group hEMT mEMT lEMT
Aucell Score
F
Positive correlations
Negative correlations
Pearson's r
G
log2 FC
abs(avg log2FC)
Significant
down
up
BC
BLCA
ccRCC
CRC
ESCC
GC
HCC
HNSCC
LC
OC
PDAC
PRAD

Figure S5. Functional characteristics of NMF programs and their associations with gene modules. (A) Heatmap showing HALLMARK pathway enrichment results for 13 NMF programs. (B) Heatmap presenting GO biological process enrichment results for 13 NMF programs. (C) Table listing the representative feature genes associated with each NMF program. (D) UMAP visualization depicting the distribution of NMF program scores across malignant epithelial cells. (E) Bar plot showing AUCell scores of the 13 NMF programs across subgroups, with statistical significance indicated (*$p < 0.05$, **$p < 0.01$, ***$p < 0.001$, ns = not significant). (F) Heatmaps (left and right) displaying correlations among WGCNA modules, with the Sankey diagram (center) illustrating associations between WGCNA modules and NMF program scores. Red and blue denote positive and negative correlations, respectively; ribbon width reflects correlation strength, and gray ribbons indicate non-significant associations ($p > 0.05$ or $|cor| < 0.1$). (G) Volcano plots showing differentially expressed genes identified between high- and low-scoring subgroups across cancer types.

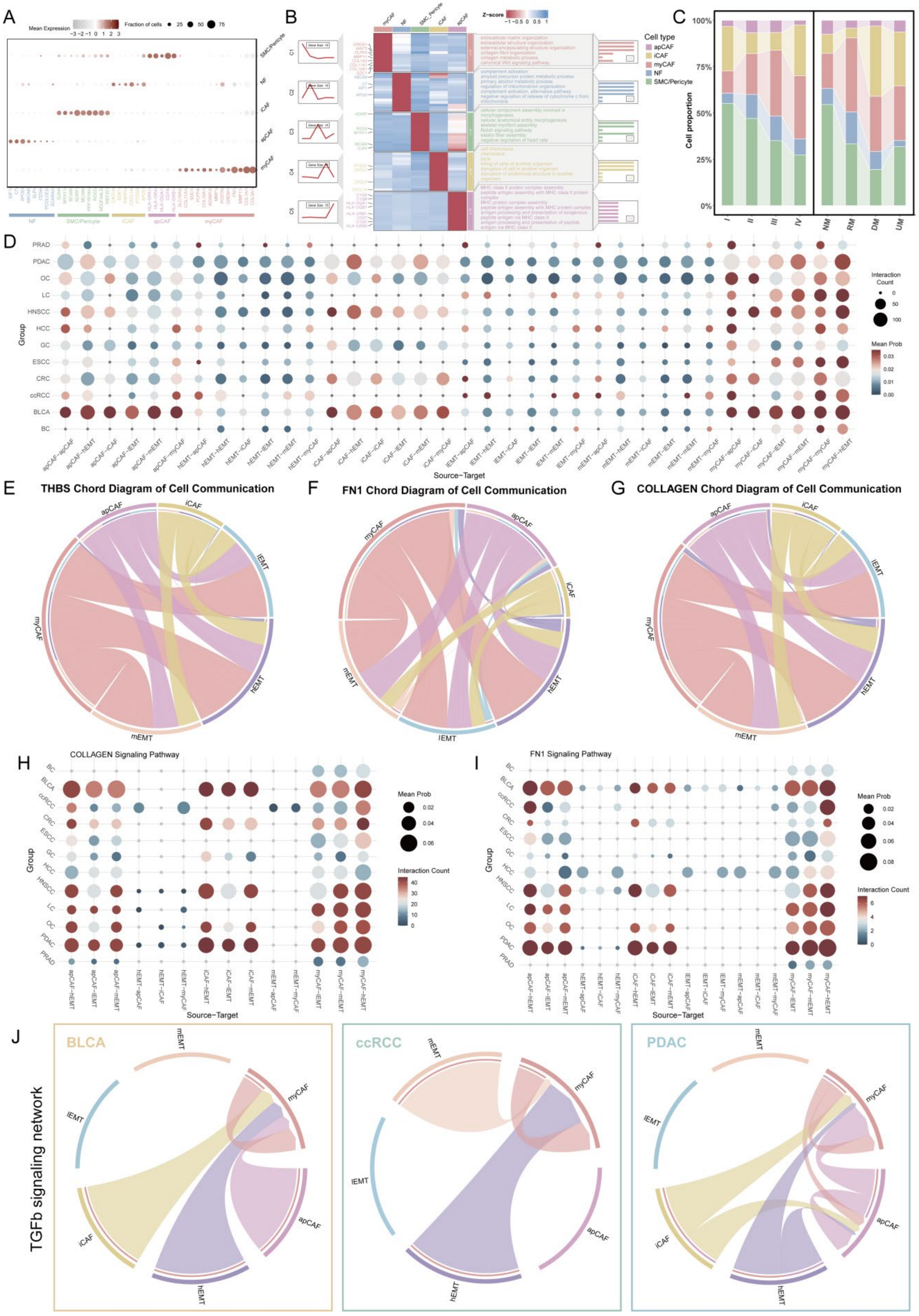
A
Mean Expression
Fraction of cells
SMC/Pericyte
NF
iCAF
apCAF
myCAF
B
Z−score
C
Cell type
apCAF
iCAF
myCAF
NF
SMC/Pericyte
Cell proportion
100%
75%
50%
25%
0%
I
II
III
IV
NM
RM
DM
UM
D
Group
PRAD
PDAC
OC
LC
HNSCC
HCC
GC
ESCC
CRC
ccRCC
BLCA
BC
Interaction Count
Mean Prob
Source−Target
E
THBS Chord Diagram of Cell Communication
F
FN1 Chord Diagram of Cell Communication
G
COLLAGEN Chord Diagram of Cell Communication
apCAF
iCAF
lEMT
hEMT
mEMT
myCAF
H
COLLAGEN Signaling Pathway
I
FN1 Signaling Pathway
J
TGFb signaling network
BLCA
ccRCC
PDAC

Figure S6. Fibroblast subtype characteristics and interactions between hEMT and myCAF cells. (A) Dot plot showing marker gene expression levels of fibroblast cell types annotated in Figure 3A, with dot color representing average expression and dot size indicating the proportion of cells expressing the gene within each cell type. (B) Composite plot illustrating marker gene expression and functional enrichment across different cell types. The line plot (left) shows expression trends of marker genes across cell populations, the heatmap (middle) depicts average expression levels of the top 10 differentially expressed genes for each cell type, and the bar plot (right) shows GO biological process (BP) enrichment results, with different colors representing cell types and bar length indicating enrichment magnitude. (C) Stacked bar plot showing proportions of each cell type across stages and metastatic states. (D) Dot plot illustrating intercellular interactions among cell types across all signaling pathways in different cancers. (E-G) Chord diagrams displaying intercellular interactions in the THBS, FN1, and COLLAGEN pathways (excluding self- and intra-type interactions), with ribbon width representing the number of interacting pairs. (H-I) Dot plots showing intercellular interactions in the FN1 and COLLAGEN signaling pathways across different cancers (excluding self- and intra-type interactions). (J) Chord diagrams depicting intercellular interactions in the TGFb signaling pathway among cell types in BLCA, ccRCC, and PDAC.

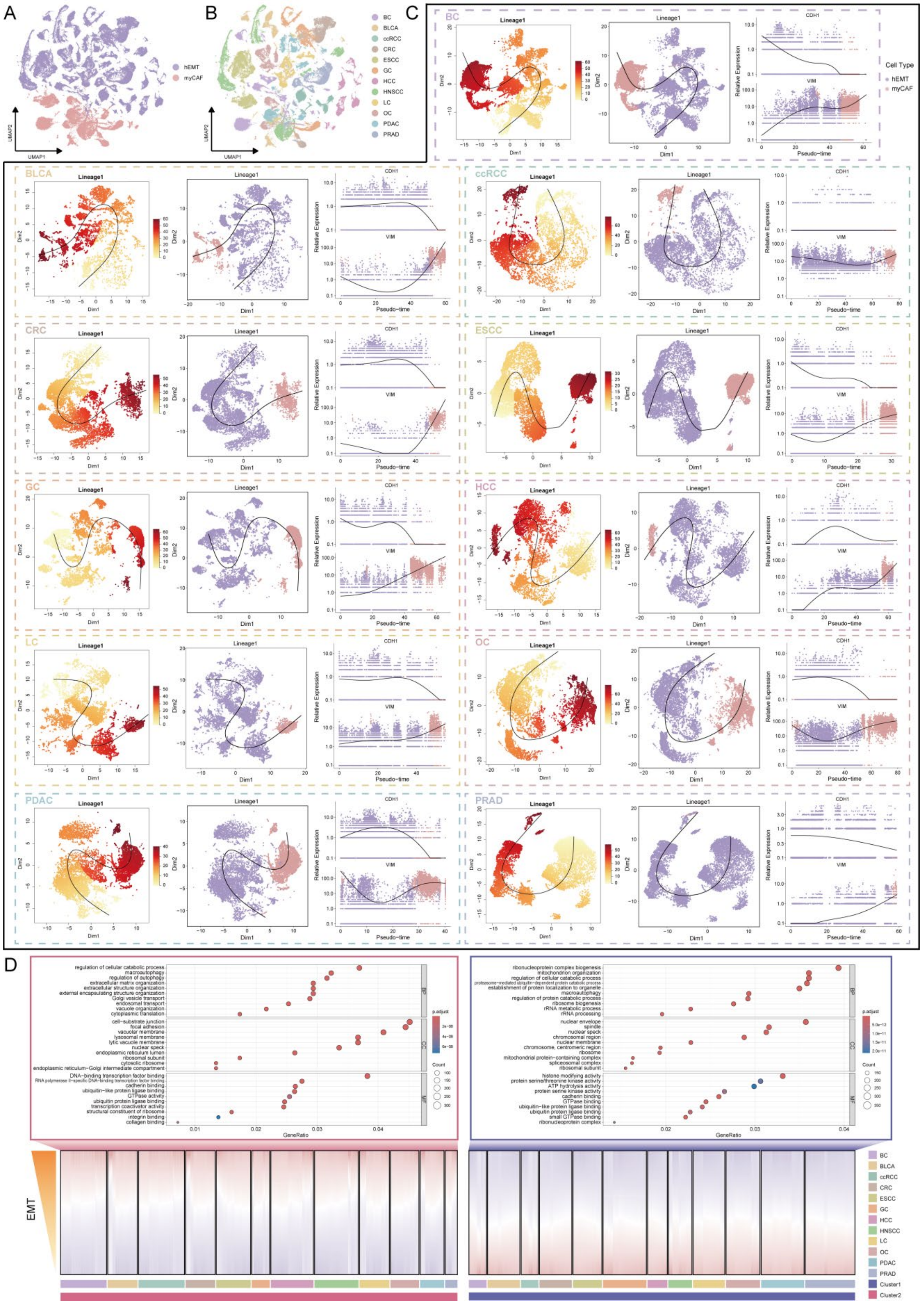
A
hEMT
myCAF
UMAP1
UMAP2
B
BC
BLCA
ccRCC
CRC
ESCC
GC
HCC
HNSCC
LC
OC
PDAC
PRAD
UMAP1
UMAP2
C
BC
BLCA
ccRCC
CRC
ESCC
GC
HCC
LC
OC
PDAC
PRAD
Lineage1
Dim1
Dim2
CDH1
VIM
Relative Expression
Pseudo-time
Cell Type
hEMT
myCAF
D
regulation of cellular catabolic process
macroautophagy
regulation of autophagy
extracellular matrix organization
extracellular structure organization
external encapsulating structure organization
Golgi vesicle transport
endosomal transport
vacuole organization
cytoplasmic translation
cell-substrate junction
focal adhesion
vacuolar membrane
lysosomal membrane
lytic vacuole membrane
nuclear speck
endoplasmic reticulum lumen
ribosomal subunit
cytosolic ribosome
endoplasmic reticulum-Golgi intermediate compartment
DNA-binding transcription factor binding
RNA polymerase II-specific DNA-binding transcription factor binding
cadherin binding
ubiquitin-like protein ligase binding
GTPase activity
ubiquitin protein ligase binding
transcription coactivator activity
structural constituent of ribosome
integrin binding
collagen binding
BP
CC
MF
p.adjust
Count
GeneRatio
ribonucleoprotein complex biogenesis
mitochondrion organization
regulation of cellular catabolic process
proteasome-mediated ubiquitin-dependent protein catabolic process
establishment of protein localization to organelle
macroautophagy
regulation of protein catabolic process
ribosome biogenesis
rRNA metabolic process
rRNA processing
nuclear envelope
spindle
nuclear speck
chromosomal region
nuclear membrane
chromosome, centromeric region
ribosome
mitochondrial protein-containing complex
spliceosomal complex
ribosomal subunit
histone modifying activity
protein serine/threonine kinase activity
ATP hydrolysis activity
protein serine kinase activity
cadherin binding
GTPase binding
ubiquitin-like protein ligase binding
ubiquitin protein ligase binding
small GTPase binding
ribonucleoprotein complex
BP
CC
MF
p.adjust
Count
GeneRatio
EMT
BC
BLCA
ccRCC
CRC
ESCC
GC
HCC
HNSCC
LC
OC
PDAC
PRAD
Cluster1
Cluster2

Figure S7. Differentiation trajectories and dynamic gene expression of hEMT and myCAF cells. (A-B) UMAP plots showing the distribution of hEMT and myCAF cells, colored by cell type (A) and cancer type (B), respectively. (C) Slingshot differentiation trajectories of hEMT and myCAF cells across 11 cancers excluding HNSCC. Black curves indicate differentiation paths. Cells are colored by pseudotime (left) or by cell type (middle). Right: dynamic expression of marker genes CDH1 and VIM along pseudotime. (D) Heatmap (bottom) showing dynamic gene expression during epithelial-mesenchymal transition along pseudotime, with two clusters: cluster1 downregulated and cluster2 upregulated during EMT progression. Bubble plot (top) illustrates GO biological process (BP), molecular function (MF), and cellular component (CC) enrichment for the two clusters; bubble size represents gene count and color indicates significance (p.adjust).

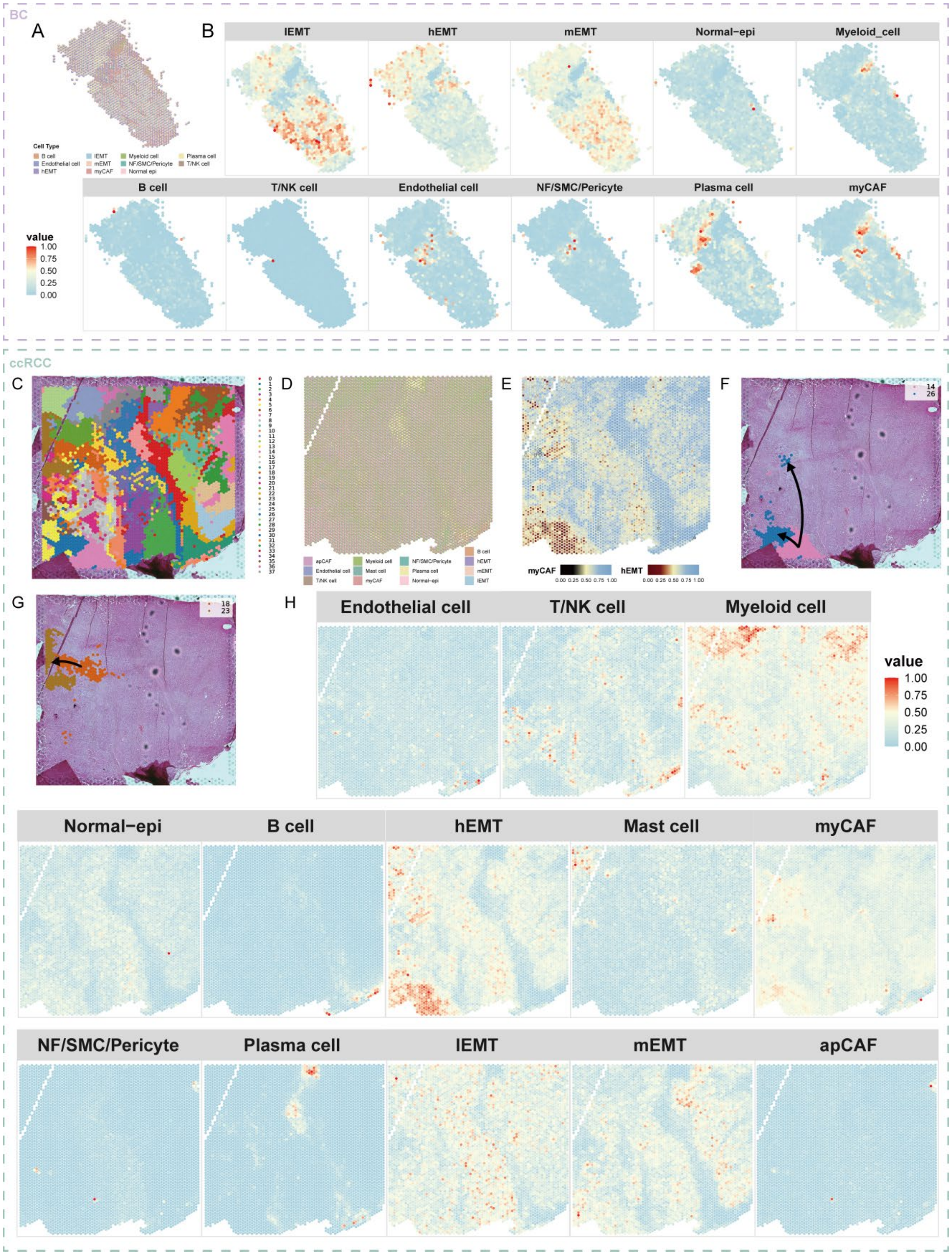

Figure S8. Spatial distribution and differentiation trajectories of hEMT and myCAF cells in spatial transcriptomics of BC and ccRCC. (A, D) Spatial composition plots showing the proportion of different single-cell types within each spot, projected from single-cell data onto spatial

transcriptomics data using the CARD method; BC (A), ccRCC (D). (B, H) Spatial feature plots depicting the predicted expression distribution of each cell type based on CARD; BC (B), ccRCC (H). (C) Subcluster plots generated by Leiden clustering in ccRCC. (E) Spatial expression patterns of hEMT cells and myCAFs in ccRCC, with myCAFs marked by black circles and hEMT cells marked by red squares. (F–G) Potential spatial differentiation trajectories of hEMT cells toward myCAF in ccRCC.

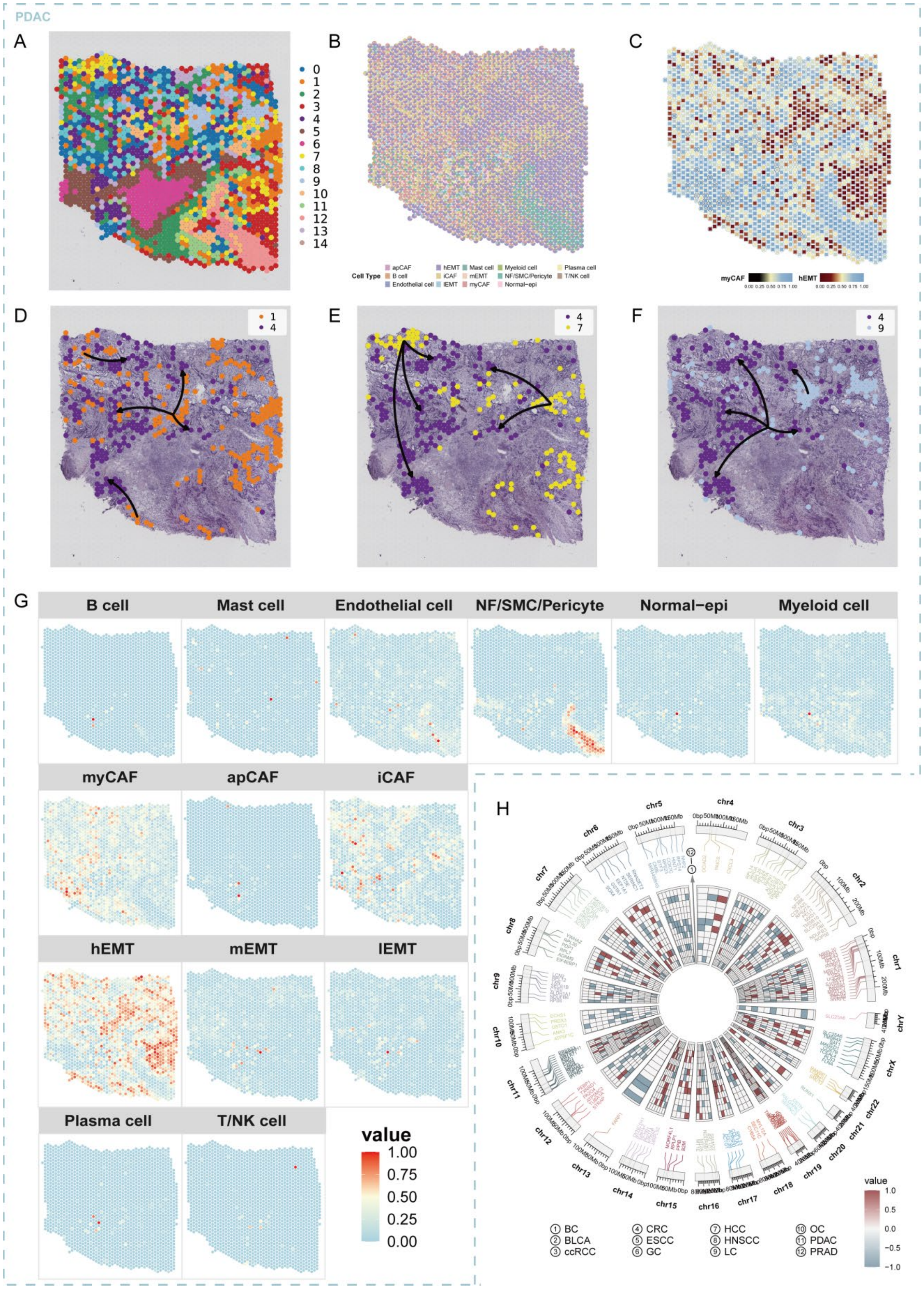
PDAC
A
0
1
2
3
4
5
6
7
8
9
10
11
12
13
14
B
Cell Type
apCAF
B cell
Endothelial cell
hEMT
iCAF
lEMT
Mast cell
mEMT
myCAF
Myeloid cell
NF/SMC/Pericyte
Normal-epi
Plasma cell
T/NK cell
C
myCAF
hEMT
0.00 0.25 0.50 0.75 1.00
D
1
4
E
4
7
F
4
9
G
B cell
Mast cell
Endothelial cell
NF/SMC/Pericyte
Normal−epi
Myeloid cell
myCAF
apCAF
iCAF
hEMT
mEMT
lEMT
Plasma cell
T/NK cell
value
1.00
0.75
0.50
0.25
0.00
H
chr1
chr2
chr3
chr4
chr5
chr6
chr7
chr8
chr9
chr10
chr11
chr12
chr13
chr14
chr15
chr16
chr17
chr18
chr19
chr20
chr21
chr22
chrX
chrY
value
1.0
0.5
0.0
−0.5
−1.0
① BC
② BLCA
③ ccRCC
④ CRC
⑤ ESCC
⑥ GC
⑦ HCC
⑧ HNSCC
⑨ LC
⑩ OC
⑪ PDAC
⑫ PRAD

Figure S9. Spatial distribution and differentiation trajectories of hEMT and myCAF cells in PDAC, and shared metastasis-signature genes. (A) Subcluster plots generated by Leiden clustering. (B) Spatial composition plots showing the proportion of different single-cell types within each spot, projected from single-cell data onto spatial transcriptomics data using the CARD method. (C) Spatial expression patterns of hEMT cells and myCAFs, with myCAFs marked by black circles and hEMT cells marked by red squares. (D–F) Potential spatial differentiation trajectories of hEMT cells toward myCAF. (G) Spatial feature plots depicting the predicted expression distribution of each cell type based on CARD. (H) Shared metastasis-signature genes across three or more cancer types. The outermost circle shows chromosomal locations, and the inner heatmap depicts co-expression patterns across cancers (red: upregulated, blue: downregulated).

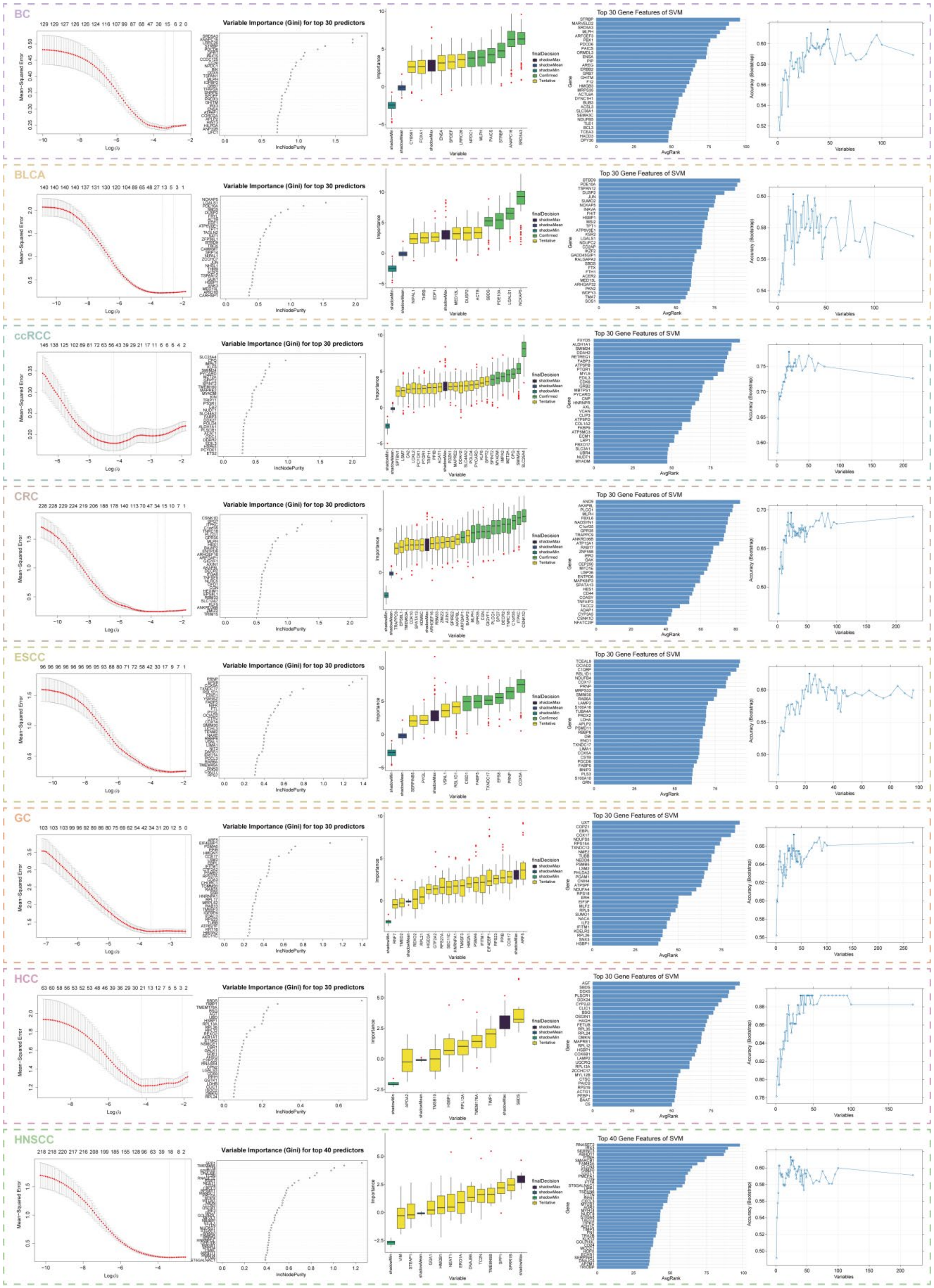
BC
BLCA
ccRCC
CRC
ESCC
GC
HCC
HNSCC
Variable Importance (Gini) for top 30 predictors
Variable Importance (Gini) for top 40 predictors
Top 30 Gene Features of SVM
Top 40 Gene Features of SVM
Mean-Squared Error
Log (λ)
IncNodePurity
Importance
Variable
finalDecision
shadowMax
shadowMean
shadowMin
Confirmed
Tentative
Gene
AvgRank
Accuracy (Bootstrap)
Variables

Figure S10. Feature selection of metastasis-related key genes across eight cancer types using multiple machine learning approaches. Results of five machine learning methods (Lasso, RF, Boruta, SVM-RFE, and RFE) applied to training datasets from BC, BLCA, and six other cancers for key gene selection. Left 1: Lasso regression cross-validation curve, with the x-axis representing log() and the y-axis representing cross-validation error; dashed lines indicate (minimum error) and (simplified model). Left 2: Random Forest variable importance dot plot (Gini index), where each point represents a gene feature's contribution to the metastasis prediction model, and its position reflects the importance magnitude. Middle: Boruta gene importance boxplot, showing the distribution of importance values across multiple Boruta iterations for each candidate gene; x-axis represents gene names, y-axis represents importance values, and box colors indicate Boruta final classification (Confirmed, Tentative, Rejected, or shadow control). Right 2: SVM top 30 gene feature ranking bar plot, with the x-axis representing gene names ordered by average rank, y-axis representing average rank (AvgRank), and bar height reflecting the mean ranking across cross-validation runs. Right: Random Forest Recursive Feature Elimination (RFE) results, with the x-axis representing the number of retained features and the y-axis representing model performance (Accuracy); the line shows performance changes with varying feature numbers, points indicate actual performance values, and solid points highlight the optimal feature subset.

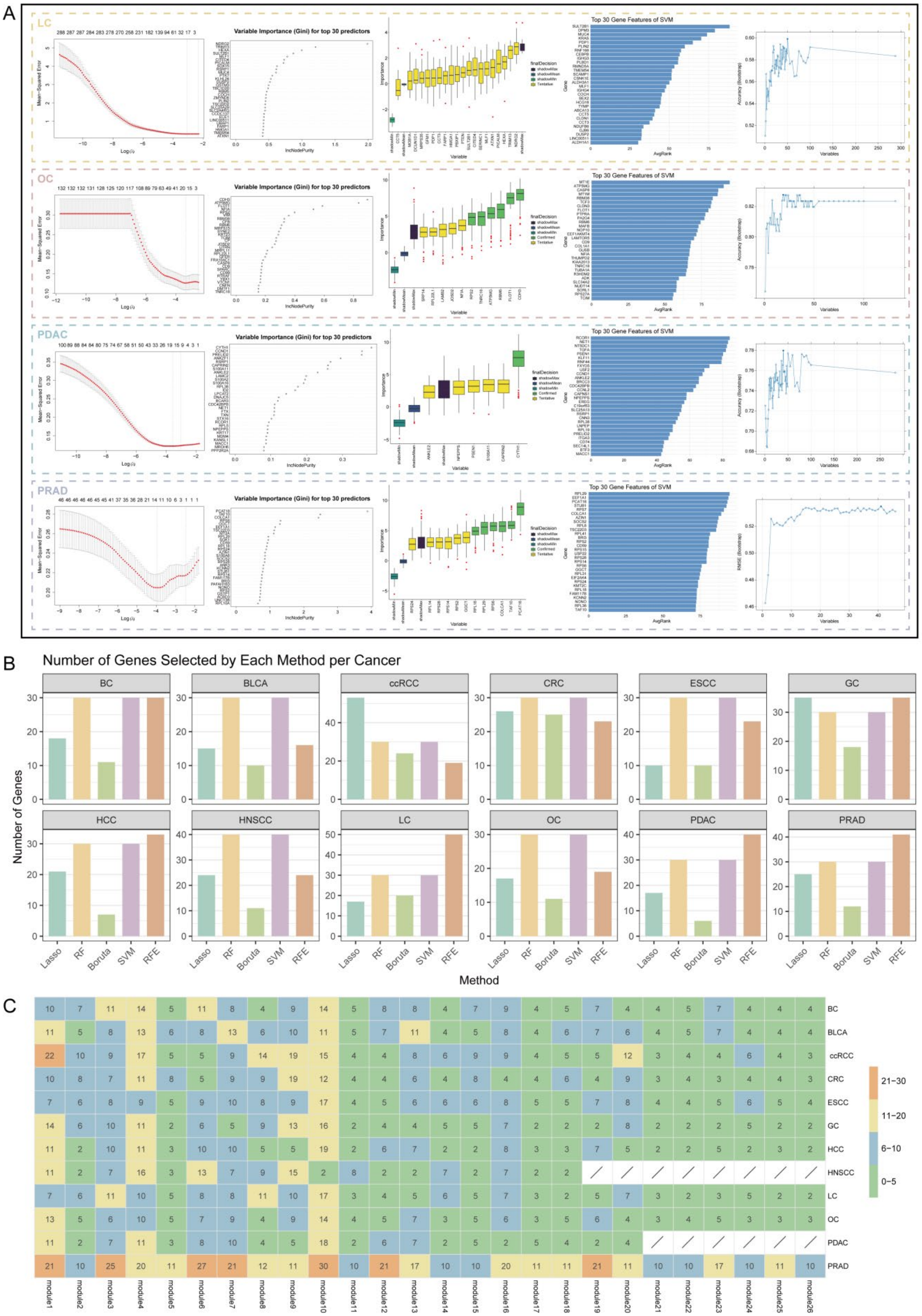
A
LC
OC
PDAC
PRAD
Variable Importance (Gini) for top 30 predictors
Top 30 Gene Features of SVM
Mean-Squared Error
Log(λ)
IncNodePurity
Importance
Variable
finalDecision
shadowMax
shadowMean
shadowMin
Confirmed
Tentative
Gene
AvgRank
Accuracy (Bootstrap)
RMSE (Bootstrap)
Variables
B
Number of Genes Selected by Each Method per Cancer
BC
BLCA
ccRCC
CRC
ESCC
GC
HCC
HNSCC
LC
OC
PDAC
PRAD
Number of Genes
Method
Lasso
RF
Boruta
SVM
RFE
C
BC: 10 7 11 14 5 11 8 4 9 14 5 8 8 4 7 9 4 5 7 4 4 5 7 4 4 4
BLCA: 11 5 8 13 6 8 13 6 10 11 5 7 11 4 5 8 4 6 7 6 4 5 7 4 4 4
ccRCC: 22 10 9 17 5 5 9 14 19 15 4 4 8 6 9 9 4 5 5 12 3 4 4 6 4 3
CRC: 10 8 7 11 8 5 9 9 19 12 4 4 6 4 8 4 4 6 4 9 3 4 3 4 4 3
ESCC: 7 6 8 9 5 9 10 8 9 17 4 5 6 6 6 8 5 5 7 8 4 4 5 6 5 4
GC: 14 6 10 11 2 6 5 9 13 16 2 4 4 5 5 7 2 2 2 8 2 2 2 5 2 2
HCC: 11 2 10 11 3 10 10 5 5 19 2 6 7 2 2 8 3 3 7 5 2 2 5 2 3 2
HNSCC: 11 2 7 16 3 13 7 9 15 2 8 2 2 7 2 7 2 2 / / / / / / / /
LC: 7 6 11 10 5 8 8 11 10 17 3 4 5 6 5 7 3 2 5 7 3 2 3 5 2 2
OC: 13 5 6 10 5 7 9 4 9 14 4 5 7 3 5 6 3 5 6 4 3 4 5 3 3 3
PDAC: 11 2 7 11 3 8 10 4 5 18 2 6 7 2 5 2 5 4 2 4 / / / / / /
PRAD: 21 10 25 20 11 27 21 12 11 30 10 21 17 10 10 20 11 11 21 11 10 10 17 10 11 10
module1 module2 module3 module4 module5 module6 module7 module8 module9 module10 module11 module12 module13 module14 module15 module16 module17 module18 module19 module20 module21 module22 module23 module24 module25 module26
21–30
11–20
6–10
0–5

Figure S11. Feature selection and summary of metastasis-related key genes across four cancer types using multiple machine learning approaches. (A) Results of five machine learning methods (Lasso, Boruta, RF, SVM, and RFE) applied to training datasets from LC, OC, and two other cancers for key gene selection (content same as Figure S10). (B) Grouped bar plot showing the number of genes selected by the five machine learning methods across different cancers. (C) Heatmap displaying the distribution of gene counts within each intersected model across different cancers, with numerical values indicated.

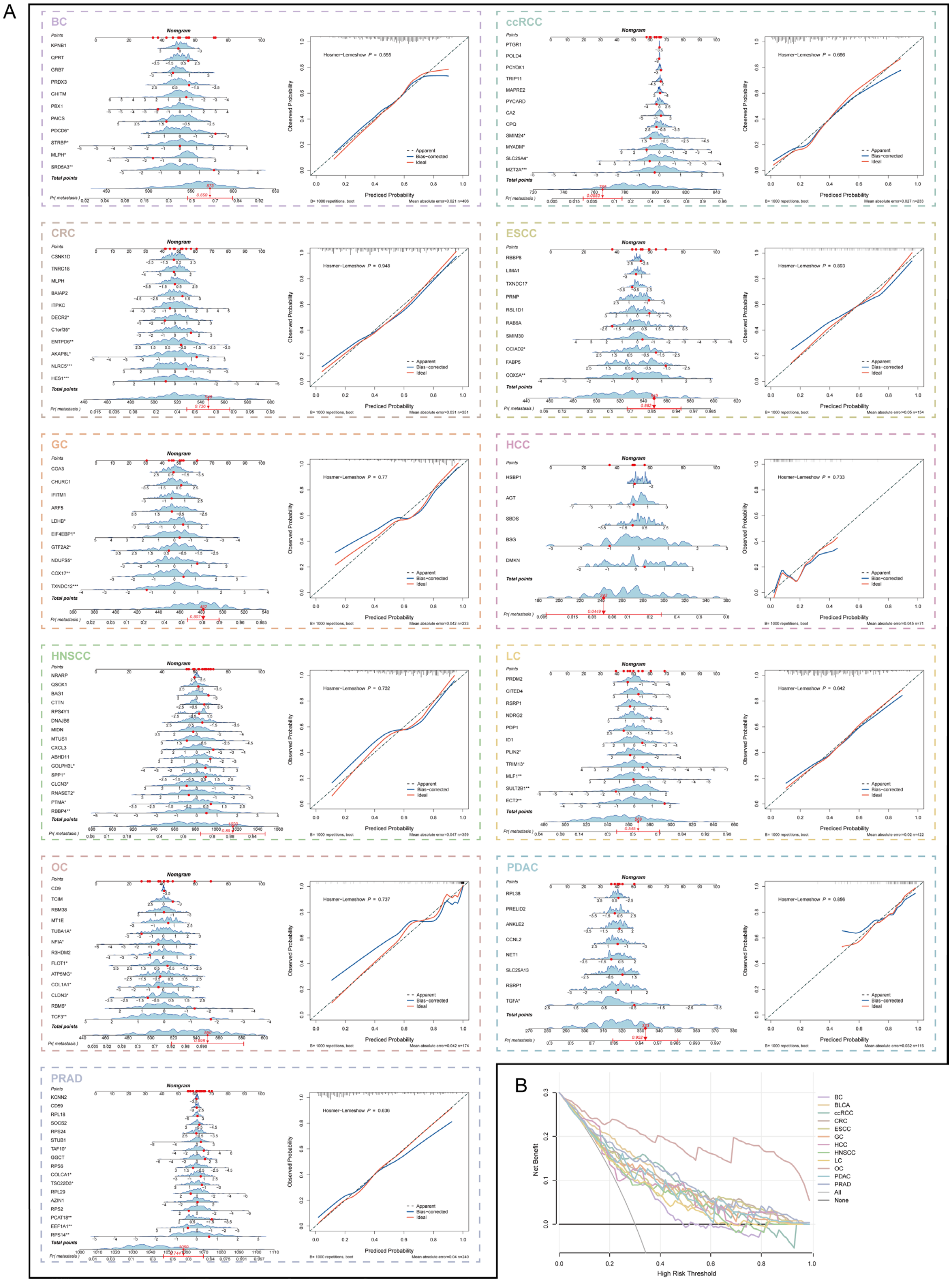

Figure S12. Construction and validation of cancer-specific metastasis prediction models. (A) Construction and validation of metastasis prediction models in 11 cancers excluding BLCA. In each cancer, the left panel shows

the nomogram for the metastasis prediction model constructed using the best model, where each row represents an individual predictive gene, each gene is assigned a corresponding score, and the total score corresponds to the patient's predicted metastasis risk. Significance indicators reflect the statistical significance of each variable in the model: * $p < 0.05$, ** $p < 0.01$, *** $p < 0.001$. The right panel shows the model calibration curve, with the x-axis representing predicted metastasis probability and the y-axis representing observed metastasis proportion. (B) Decision curve analysis (DCA) for metastasis risk prediction based on MPS across different cancers. The x-axis represents threshold probability, and the y-axis represents net benefit. Curves include model prediction (MPS), the all-intervention strategy (All), and the no-intervention strategy (None).

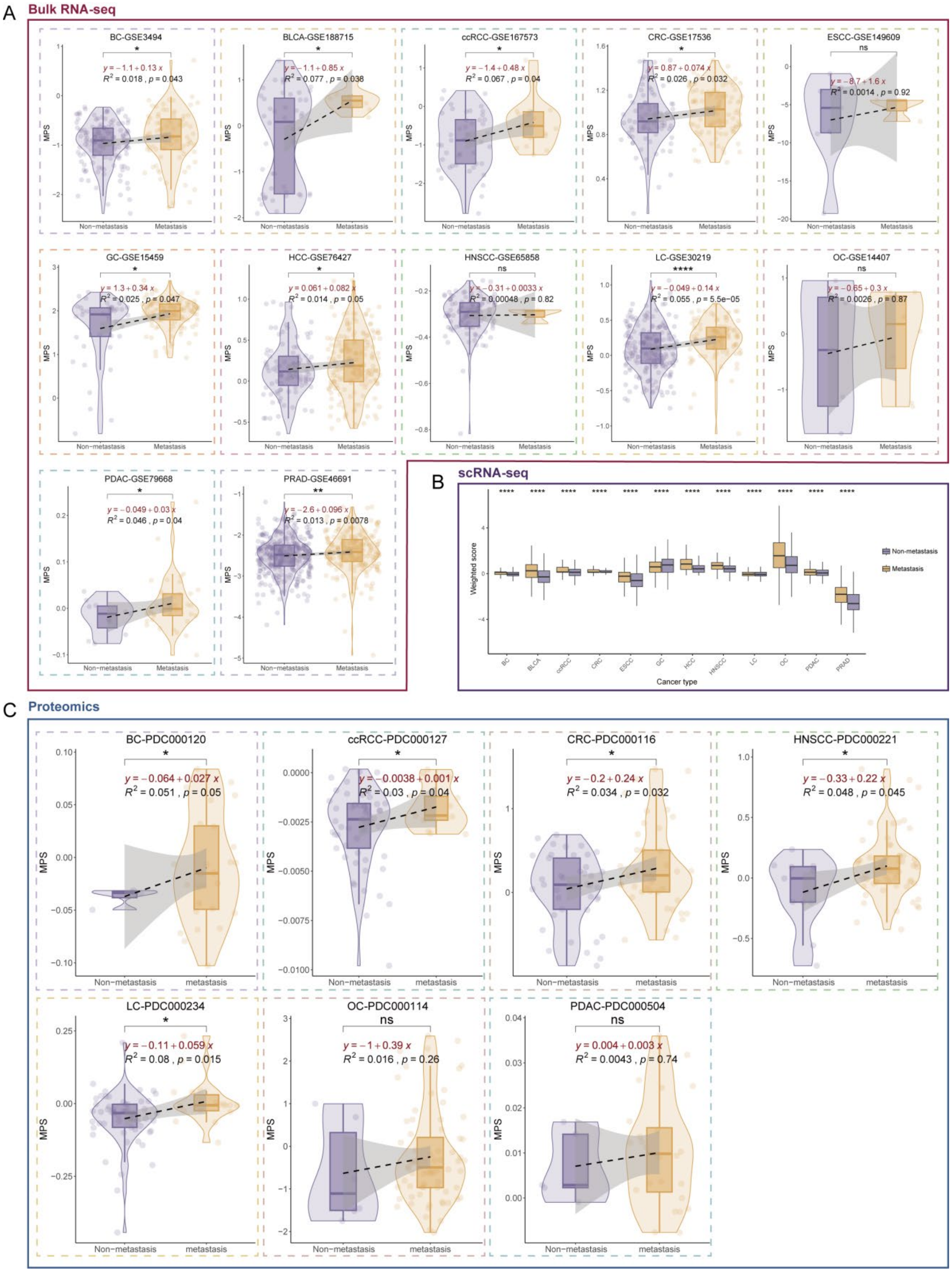

Figure S13. Validation of MPS. Boxplots showing the distribution of MPS across samples with different metastasis statuses in external bulk RNA-seq datasets (A), scRNA-seq datasets (B), and proteomics datasets (C), with

significance levels indicated (*p < 0.05, **p < 0.01, ***p < 0.001, ****p < 0.0001, ns: not significant).

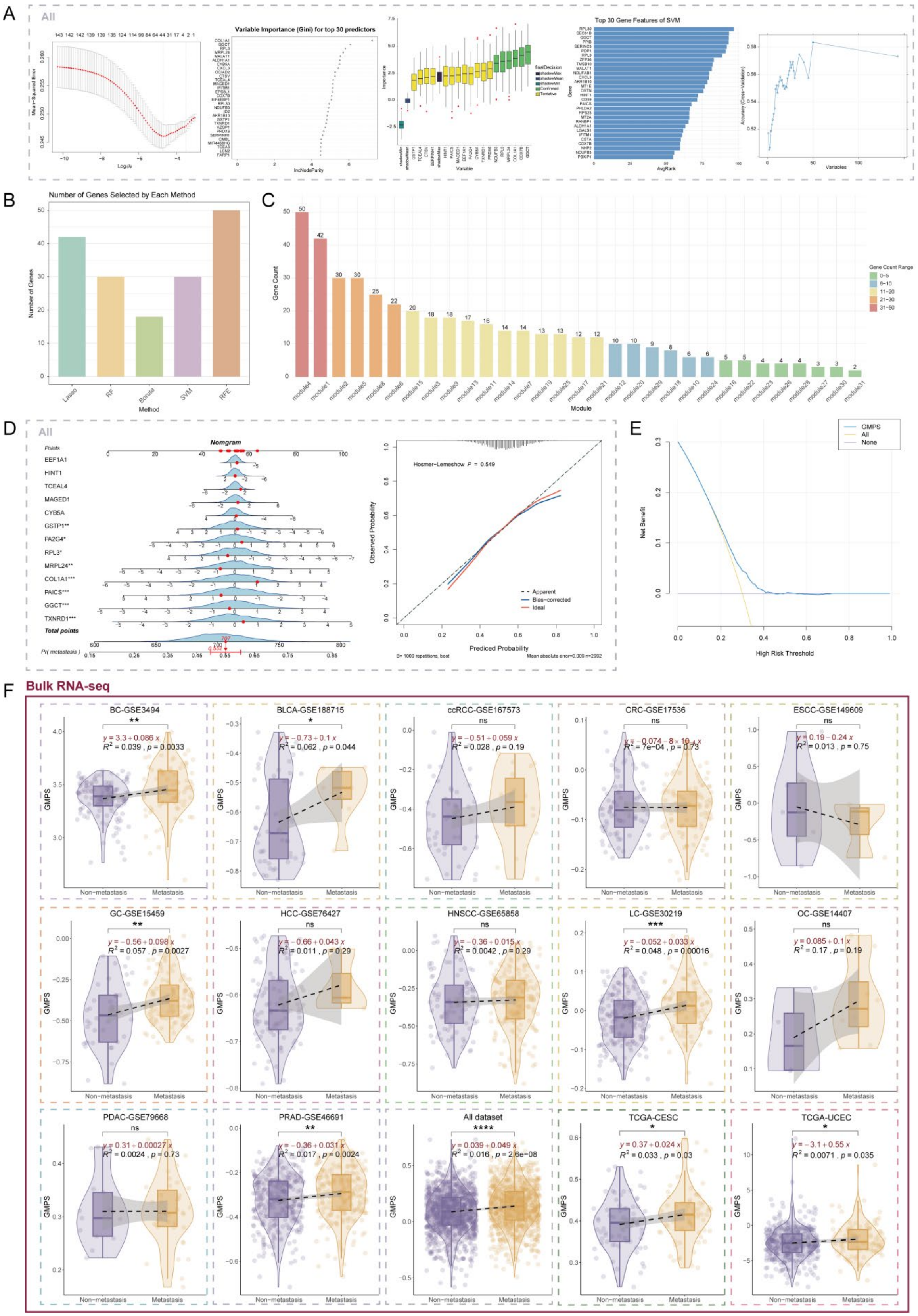
A
All
Log(λ)
Mean-Squared Error
Variable Importance (Gini) for top 30 predictors
IncNodePurity
Importance
Variable
finalDecision
shadowMax
shadowMean
shadowMin
Confirmed
Tentative
Top 30 Gene Features of SVM
Gene
AvgRank
Accuracy (Cross-Validation)
Variables
B
Number of Genes Selected by Each Method
Number of Genes
Method
Lasso
RF
Boruta
SVM
RFE
C
Gene Count
Module
Gene Count Range
0~5
6~10
11~20
21~30
31~50
D
All
Points
Nomgram
EEF1A1
HINT1
TCEAL4
MAGED1
CYB5A
GSTP1**
PA2G4*
RPL3*
MRPL24**
COL1A1***
PAICS***
GGCT***
TXNRD1***
Total points
Pr(metastasis)
Hosmer−Lemeshow P = 0.549
Observed Probability
Prediced Probability
Apparent
Bias−corrected
Ideal
B= 1000 repetitions, boot
Mean absolute error=0.009 n=2992
E
GMPS
All
None
Net Benefit
High Risk Threshold
F
Bulk RNA-seq
BC-GSE3494
BLCA-GSE188715
ccRCC-GSE167573
CRC-GSE17536
ESCC-GSE149609
GC-GSE15459
HCC-GSE76427
HNSCC-GSE65858
LC-GSE30219
OC-GSE14407
PDAC-GSE79668
PRAD-GSE46691
All dataset
TCGA-CESC
TCGA-UCEC
GMPS
Non-metastasis
Metastasis

Figure S14. Construction and validation of the global metastasis prediction model. (A) Detailed results of key gene selection in pan-cancer training datasets using five machine learning methods (Lasso, Boruta, RF, SVM, and RFE) (content same as Figure S10). (B) Bar plot showing the number of genes selected by each of the five machine learning methods in pan-cancer analysis. (C) Bar plot showing the distribution of gene numbers in each intersection model across cancers, with numerical values indicated. (D) Construction and validation of the global metastasis prediction model (content same as Figure S12A). (E) Decision curve analysis (DCA) for metastasis risk prediction based on the GMPS. (content same as Figure S12B). (F) Boxplots showing the distribution of GMPS across samples with different metastasis statuses in external bulk RNA-seq datasets, with significance levels indicated (*$p < 0.05$, **$p < 0.01$, ***$p < 0.001$, ****$p < 0.0001$, ns: not significant).

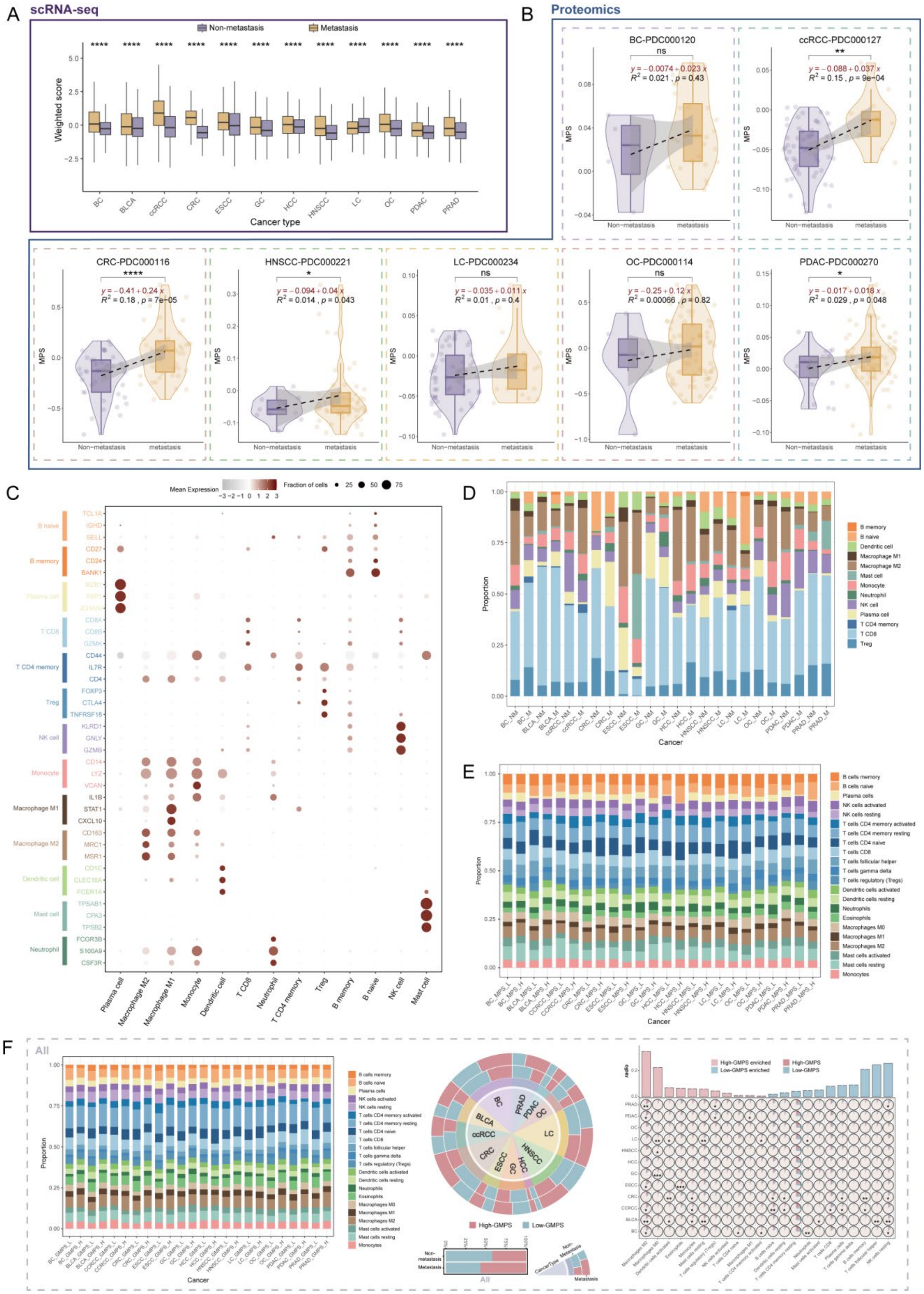

Figure S15. Validation of GMPS and immune infiltration analysis. (A-B) Boxplots showing the distribution of MPS across samples with different

metastasis statuses in scRNA-seq datasets (A), and proteomics datasets (B), with significance levels indicated (*$p < 0.05$, **$p < 0.01$, ***$p < 0.001$, ****$p < 0.0001$, ns: not significant). (C) Dot plot showing the expression levels of marker genes for each cell type annotated in Figure 4F; dot color represents the average expression level, and dot size represents the proportion of cells in which the gene is expressed within a specific cell type. (D) Stacked bar plots showing the proportions of immune cells in metastasis and non-metastasis samples across different cancers in single-cell data. (E) Stacked bar plots illustrating immune infiltration in different MPS risk groups across cancers in RNA-seq data. (F) Left: stacked bar plots showing immune infiltration in different GMPS risk groups across cancers in RNA-seq data; middle: circular bar plots showing the proportions of High-GMPS and Low-GMPS samples in each cancer type; right: immune infiltration landscape of different GMPS risk groups in RNA-seq data. The heatmap (bottom) shows differences in immune cell subset proportions between High-GMPS and Low-GMPS groups and their significance (*$p < 0.05$, **$p < 0.01$, ***$p < 0.001$); the bar plot (top) shows relative changes of each cell type between the two groups, with colors indicating group enrichment.

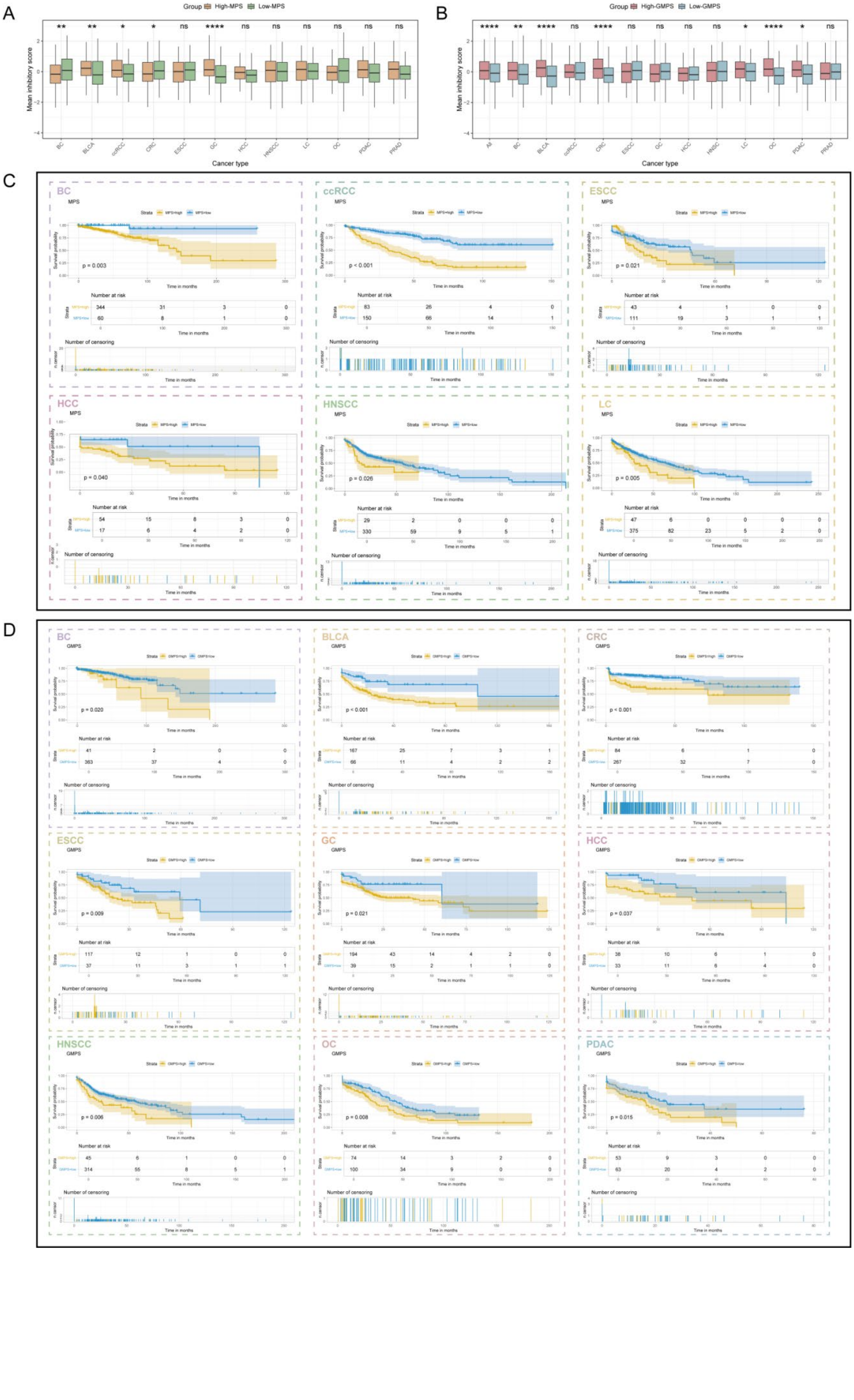
A
Group High-MPS Low-MPS
Mean inhibitory score
Cancer type
BC BLCA ccRCC CRC ESCC GC HCC HNSCC LC OC PDAC PRAD
** ** * * ns **** ns ns ns ns ns ns
B
Group High-GMPS Low-GMPS
Mean inhibitory score
Cancer type
All BC BLCA ccRCC CRC ESCC GC HCC HNSC LC OC PDAC PRAD
**** ** **** ns **** ns ns ns ns * **** * ns
C
BC
MPS
p = 0.003
ccRCC
MPS
p < 0.001
ESCC
MPS
p = 0.021
HCC
MPS
p = 0.040
HNSCC
MPS
p = 0.026
LC
MPS
p = 0.005
D
BC
GMPS
p = 0.020
BLCA
GMPS
p < 0.001
CRC
GMPS
p < 0.001
ESCC
GMPS
p = 0.009
GC
GMPS
p = 0.021
HCC
GMPS
p = 0.037
HNSCC
GMPS
p = 0.006
OC
GMPS
p = 0.008
PDAC
GMPS
p = 0.015
Number at risk
Number of censoring
Time in months
Survival probability
Strata

Figure S16. Immune checkpoint scoring and survival analysis. (A-B) Boxplots showing the distribution of inhibitory immune checkpoint scores in different MPS (A) and GMPS (B) risk groups, with significance levels indicated (*$p < 0.05$, **$p < 0.01$, ***$p < 0.001$, ****$p < 0.0001$, ns: not significant). (C-D) Association of MPS with survival in six cancers including BC and ccRCC (C), and association of GMPS with survival in nine cancers including BC and BLCA (D). Kaplan-Meier curves show the survival probability of patients with high and low gene expression over time (months), with the number of at-risk and censored patients indicated below the curves; shaded areas represent confidence intervals. Survival differences between groups are significant ($p < 0.05$).

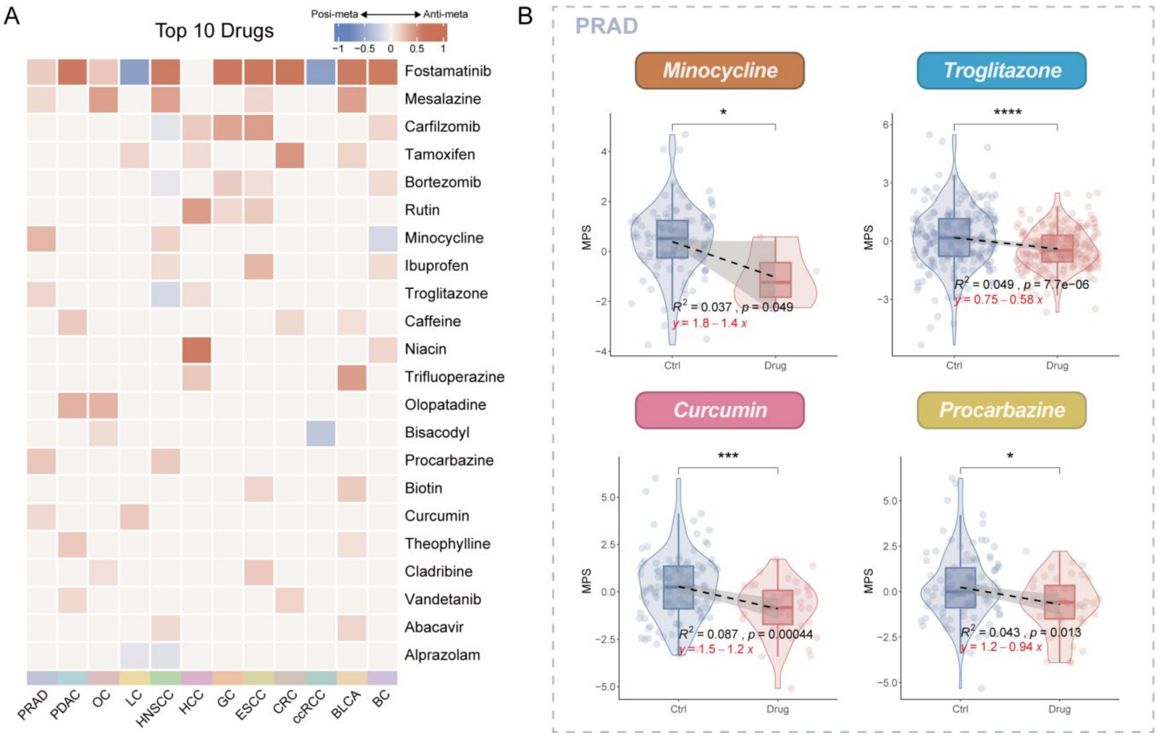

Figure S17. Shared drugs and model validation. (A) Heatmap analysis of the top 10 candidate drugs shared across two or more cancer types. The color intensity represents the magnitude of the Drug Score, while the color gradient indicates the regulatory effect on metastasis (red: inhibition; blue: promotion). (B) Boxplots comparing MPS between treated and control groups for Minocycline, Troglitazone, Curcumin, and Procarbazine in PRAD.
